# Supplementary material for: Mesoporous Organosilica Nanoparticles to Fight Intracellular Staphylococcal Aureus Infections in Macrophages
Source: Pharmaceutics. 2023 Mar 23;15(4):1037. doi: 10.3390/pharmaceutics15041037 (PMC10146421; doi:10.3390/pharmaceutics15041037)
Supplement: Supplementary file 1 [file pharmaceutics-15-01037-s001.zip › pharmaceutics-2235411-supplementary.pdf]

Mesoporous organosilica nanoparticles to fight intracellular *Staphylococcal aureus* infections in macrophages

Manasi Jambhrunkar[1,2]*, Sajedehsadat Maghrebi*[1]*, Divya Doddakyathanahalli*[1]*, Anthony Wignall*[1]*, Clive A. Prestidge*[1,2#] *and Kristen E. Bremmell*[1#*]

[1]Centre for Pharmaceutical Innovation, UniSA Clinical and Health Sciences, University of South Australia, Adelaide, South Australia 5000, Australia

Supporting information

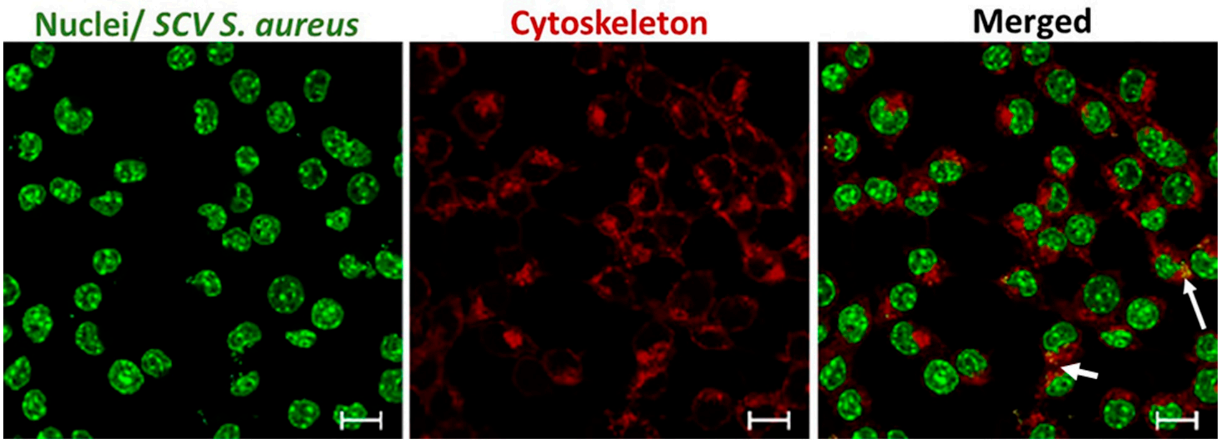

Figure S1: CSLM images of RAW 264.7 cells infected with SCV *S. aureus*; Nuclei and SCV S. aureus were stained with DAPI dye (green), the cellular cytoskeleton were stained with Phalloidin Alexa 488 (red). The white arrows depict the intracellular SCV *S. aureus*.
